# Do Fat Supplements Increase Physical Performance?

**DOI:** 10.3390/nu5020509

**Published:** 2013-02-07

**Authors:** Filippo Macaluso, Rosario Barone, Patrizia Catanese, Francesco Carini, Luigi Rizzuto, Felicia Farina, Valentina Di Felice

**Affiliations:** Department of Experimental Biomedicine and Clinical Neurosciences, University of Palermo, Via del Vespro, 129, Palermo, Italy; E-Mails: filippo.macaluso@unipa.it (F.M.); rusbarone@hotmail.it (R.B.); patriziacatanese@libero.it (P.C.); francesco.carini@unipa.it (F.C.); luigirizzuto@hotmail.com (L.R.); felicia.farina@unipa.it (F.F.)

**Keywords:** fish oil, conjugated linoleic acid, endurance exercise, resistance exercise, steroidogenic cells, steroidogenesis, testosterone

## Abstract

Fish oil and conjugated linoleic acid (CLA) belong to a popular class of food supplements known as “fat supplements”, which are claimed to reduce muscle glycogen breakdown, reduce body mass, as well as reduce muscle damage and inflammatory responses. Sport athletes consume fish oil and CLA mainly to increase lean body mass and reduce body fat. Recent evidence indicates that this kind of supplementation may have other side-effects and a new role has been identified in steroidogenensis. Preliminary findings demonstrate that fish oil and CLA may induce a physiological increase in testosterone synthesis. The aim of this review is to describe the effects of fish oil and CLA on physical performance (endurance and resistance exercise), and highlight the new results on the effects on testosterone biosynthesis. In view of these new data, we can hypothesize that fat supplements may improve the anabolic effect of exercise.

## 1. Introduction

Many food supplements claim to induce weight loss by increasing lean body mass or reducing body fat mass, although only a few of these ergogenic aids have been investigated [1]. This review focuses on a popular class of food supplements known as “fat supplements”, which are marketed with claims to induce weight loss, alter lipid profiles, improve performance, increase fat metabolism and spare glycogen stores during endurance exercise [2].

The class of commercially available fat supplements includes conjugated linoleic acid (CLA), fish oil, long- and medium-chain triacylglycerols. These ergogenic aids are claimed to be associated with a reduction in muscle glycogen breakdown, improved endurance capacity, reduced body mass and a reduction in muscle damage and inflammatory responses [2]. Only two fat supplements have been shown to affect testosterone biosynthesis: fish oil and CLA.

Fish oil contains both the omega-3 fatty acids docosahexaenoic acid (DHA) and eicosapentaenoic acid (EPA). They are polyunsaturated fatty acids (PUFA) with a double carbon bond starting after the third carbon atom from the end of the carbon chain [3]. The main source of omega-3 fatty acids is fish (such as tuna and salmon), although fish do not produce omega-3 fatty acids, they accumulate them by consuming either microalgae or fish that have a large quantity of omega-3 fatty acids. Food integration with DHA and EPA seems to reduce the incidence of cardiovascular diseases [4,5], reduce the release of inflammatory acute-phase proteins [6,7], and reduce superoxide anion production from stimulated blood neutrophils [8], although an old epidemiologic study stated that fish oil did not lower the risk of cardiovascular disease [9].

The CLA supplement is a mixture of positional and geometrical conjugated dienoic isomers of linoleic acid which present two double bonds separated by a single bond [10]. These double bonds can be located in any position of the carbon chain, commonly between 8 and 13, and in a cis or trans configuration. The two most common isomers of CLA are cis-9, trans-11 and trans-10, cis-12 (c9:t11 and t10:c12, respectively) [10,11]. The major sources of CLA in human diets are ruminant meats (beef and lamb) and dairy products (milk and cheese) [12]. It has been reported that CLA has anti-obesity potentials, such as decreasing lipogenesis and food intake, and increasing energy expenditure, lipolysis and fat oxidation [13,14].

Recent results suggest a new role of this class of supplementation in testosterone biosynthetic pathways. This review describes the various fat supplements pointing out both the known effects produced by these supplements when associated with exercise, and the new data underlying the molecular mechanisms regulating testosterone biosynthesis. Finally, it is briefly described how these fat supplements may influence physical performance. This review focuses mainly on human studies, although animal and *in vitro* studies have been cited whenever the information is not available in humans. 

**Figure 1:**
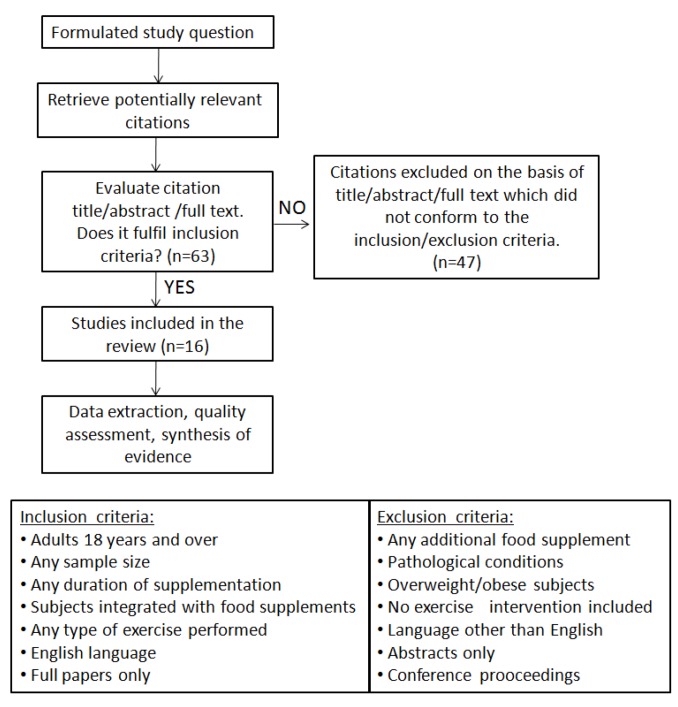
Methodological procedure of literature research.

Potential studies were identified by searching electronic databases: PubMed, Cochrane, and Scopus. The search terms used included both single words and combinations of words: CLA, fish oil, testosterone, exercise. Bibliographies were checked and experts were consulted for any additional studies. Studies available as full papers were deemed eligible if they conformed to the predetermined inclusion and exclusion criteria (Figure 1).

## 2. Association of Exercise and Fat Supplements

Elite and recreational athletes, who participate in various types of physical activity and sports, consume fish oils and CLA supplements to improve their performance, increase training effects, reduce body fat, increase lean body mass, and reduce muscle damage and inflammatory responses. The following paragraph summarizes the main results obtained from trained individuals after integration with fish oil (Table 1) or CLA (Table 2), comparing these results with the ones obtained in animal studies. All of the studies that have investigated the effects of these fat supplements in combination with other food supplements have not been cited, since it was impossible to isolate the single effect of fish oil or CLA.

Only a few studies have examined whether fish oil supplementation during training enhances endurance adaptations. These studies conducted in humans show controversial results, but in our opinion this is due to the difference in the level of training of the study participants. In elite or well-trained athletes, the margin of improvement is so inappreciable that it would not be a surprise if small differences in performance parameters were observed; while in sedentary subjects, starting a training program, the improvement in performance is considerable, making a possible small enhancement in performance induced by food supplementation undetectable. Therefore the most reliable results have been observed in trained subjects, who already show adaptations for that specific type of exercise, although still improving their performance.

**Table 1:** Effects of fish oil supplementation associated with exercise.

Reference	Study Design	Participants (N, sex)	Exercise interventions	Time (fish oil)	Main outcome
Oostenbrug *et al.* [15]	D–R–P	Cyclist (24, M)	Acute aerobic bout (60 min time trial)	3 weeks (6 g/day)	• No effect: endurance performance
Buckley *et al.* [16]	D–R–P	Australian rules football players (25, M)	Acute aerobic bout to exhaustion	5 weeks (6 g/day)	• No effect: endurance performance, recovery;
					• Improve: CV function
Raastad *et al.* [17]	D–R–P	Soccer players (28, M)	Routine training (not supervised)	10 weeks (5.2 g/day)	• No effect: maximal aerobic power, anaerobic power, performance
Peoples *et al.* [18]	D–R–P	Cyclist (16, M)	Acute aerobic bout (50% of peak workload)	8 weeks (8 g/day)	• No effect: endurance performance;
					• Reduce: whole-body and myocardial O_2_ demand
Brilla *et al.* [19]	D–R	Sedentary (32, M)	60 min (3 day/week) aerobic exercise	10 weeks (4 g/day)	• No effect: Body composition;
					• Improve: VO_2max_, VAT
Guezennec *et al.* [20]	D–R–P	Healthy (14, M)	Acute aerobic bout (60 min 70% of VO_2max_)	6 weeks (6 g/day)	• Improve: VO_2max_, RBC deformability
Ernst *et al.* [21]	D	Healthy (14, M)	Acute aerobic bout	3 weeks (2.8 g/day)	• Reduce: inflammatory acute-phase response
Toft *et al.* [22]	D–R	Runners (20, M)	Marathon	6 weeks (2.8 g/day)	• No effect: inflammatory acute-phase response
Lenn *et al.* [23]	D–R–P	Healthy (22, M)	50 Maximal eccentric elbow flexion contractions	30 days (1.8 g/day)	• No effect: inflammatory acute-phase response

Abbreviations present in the table: D, double-blind; R, randomised; P, placebo-controlled; M, male; CV, cardiovascular; VAT, ventilatory aerobic threshold; RBC, red blood cells.

**Table 2:** Effects of CLA supplementation associated with exercise.

Reference	Study Design	Participants (N, sex)	Exercise interventions	Time (CLA)	Main outcome
Zambell *et at.*[14]	D–R–P	Healthy (17, F)	Acute aerobic bout (walking)	64 days (3 g/day)	• No effect: energy expenditure, RER, Fat oxidation
Kreider *et al.* [24]	D–R–P	Bodybuilders (23, M)	Resistance training(not supervised)	4 weeks (6 g/day)	• No effect: Body composition, bone density, strength
Lambert *et al.* [25]	D–R–P	Physically active (25, M; 37, F)	Routine training (not supervised)	12 weeks (3.9 g/day)	• No effect: Body composition, RER
Macaluso *et al.* [26]	D–R–P–C	Physically active (10, M)	Resistance training + Acute resistance bout	3 weeks (6 g/day)	• No effect: Body composition;
					• Slight increase total testosterone
Thom *et al.* [27]	D–R–P	Physically active (10, M; 10, F)	90 min (3 day/week)Strenuous exercise	12 weeks (1.8 g/day)	• Improve: Body composition, endurance performance
Colakoglu *et al.* [28]	D–R–P–C	Healthy (44, F)	30 min (3 day/week)Aerobic exercise	6 weeks (3.6 g/day)	• Improve: Body composition, endurance performance
Pinkoski *et al.* [29]	D–R–P	Healthy (17, F)	90 min (3 day/week)Resistance exercise	7 weeks (5 g/day)	• Improve: Body composition

Abbreviations present in the table: CLA, conjugated linoleic acid; D, double-blind; R, randomised; P, placebo-controlled; C, crossover; M, male; F, female; RER, respiratory exchange ratio.

One of the effects claimed by fish oil is the ability to modify the viscosity of the plasma membrane of red blood cells (RBC), improving their deformability when they pass through the capillary bed [30,31,32]. Alterations in lipid membrane physical features probably depend on the integration and enrichment of omega-3 fatty acids [32]. The first studies were performed on healthy humans [30] and angina patients [31], later on it was hypothesized that, in the same way, fish oil could enhance oxygen delivery to contracting muscle and maximum oxygen uptake (VO_2max_), thus improving endurance performance [2]. It was observed that endurance exercise itself increases the fraction of PUFA in muscle membranes [33].

However, literary data are controversial. Oostenbrug *et al.* [15] studied the effects of three weeks fish oil feeding (6 g/day) and observed a small not significant decrease (2%) in RBC deformability, which appears unlikely to affect VO_2max_ or exercise performance of well-trained cyclists [15]. Others observed that fish oil supplementation does not improve exercise performance of elite athletes practicing different sports (elite Australian rules footballers [16], well-trained soccer players [17]). Fish oil reduces both whole-body and myocardial oxygen demand during exercise, without affecting performance [18]. Sedentary males supplemented with fish oil for 10 weeks (4 g/day) and exercised three times per week, had no additional effect on VO_2max_ compared to only training effect, although the supplemented exercised subjects and the supplemented non-exercised subjects showed an increase in the ventilatory aerobic threshold compared to the control [19]. 

The most significant documented results were observed in fit male subjects supplemented with fish oil (6 g/day) for six weeks [20]. Fish oil feeding increased the fraction of omega-3 fatty acids in RBC membranes, increasing their deformability during hypobaric exercise; VO_2max_ increased significantly and O_2_ desaturation rate decreased as an effect of fish oil supplementation [20].

The extensive literature on the effect of omega-3 supplementation also provides evidence that fish oil is effective in the prevention and treatment of inflammatory conditions [34]. It was hypothesized that fish oil supplementation may prevent secondary muscle damage induced by an acute inflammatory response in reaction to tissue damage caused by a bout of intense exercise. Inflammation and inflammatory responses are maintained in the muscle tissue by local and systemic elevation of specific cytokines, such as tumor necrosis factor-α (TNF-α) and interleukin-6 (IL-6). Controversial results are reported in the literature on the effects of fish oil supplementation in reducing the inflammatory response and delayed-onset muscle soreness, following acute exercise. Ernst *et al.* [31] have shown a reduction in the rise of acute-phase proteins, associated with the inflammatory response, in healthy males after three weeks of omega-3 supplementation (3.80 g/day). No difference in blood levels of TNF-α and IL-6 were observed in runners after a marathon run and in sedentary subjects following maximal isokinetic eccentric elbow-flexor contractions supplemented with fish oil (3.6 g/day for six weeks, 1.8 g/day for six weeks; respectively) [22,23]. Nieman *et al.* [35] also showed that *n*-3 PUFA somministration did not alter inflammatory proteins and plasma cytokines. The results on secondary muscle damage induced by an acute inflammatory response are extremely interesting, and more research is needed before conclusions can be drawn on fish oil supplementation in trained individuals. Omega-3 supplementation may provide benefits by minimizing the recovery time between exercise sessions reducing the inflammatory response localized in muscle tissue as well as the associated delayed-onset of muscle soreness.

CLA supplementation may induce a reduction in body weight, this statement is based on results obtained in humans and in animals, even if the effect on humans is less clear than in animals. In many studies CLA supplementation was not associated with any regular and supervised physical activity. Only six studies have been conducted to evaluate the effect of CLA supplementation associated with exercise. Kreider *et al.* [24] investigating the effects of CLA supplementation for four weeks (6 g/day) in bodybuilders concluding that CLA does not appear to possess any significant ergogenic value, since no differences were observed in body composition and strength at the end of the supplementation period. No change in body composition was observed by Lambert *et al.* [25] after CLA supplementation. The study was conducted using regular non-obese exercising men and woman integrated with CLA (3.9 g/day) for 12 weeks. Similar results have been reported by our research group, in a study performed with regularly exercising men integrated with CLA (6 g/day) for four weeks [26]. On the contrary other studies demonstrated a significant reduction in body fat but not body weight in men upon CLA supplementation. The study of Thom *et al.* [27] where men and women were supplemented with 1.8 g/day CLA for 12 weeks combined with a standardized physical exercise protocol of 90 min three times per week, and the study of Colakoglu *et al.* [28] showed that both 3.6 g/day CLA for six weeks and exercise (30 min, 3 days per week, for 6 weeks) are effective in improving endurance performance and body composition. A small effect was determined by Pinkoski *et al.* [29] who studied the effects of CLA supplementation during resistance exercise; they performed a cross over study where subjects were randomized to receive CLA (5 g/day) or placebo for seven weeks while performing resistance training three days per week.

We can conclude that CLA supplementation, associated with resistance training, results in an increase in lean body mass and a decrease in body fat mass only when the subjects are involved in standardized and supervised exercise sessions during the supplementation period.

## 3. Testosterone Biosynthesis

In males testosterone is mainly (>95%) synthesized in Leydig cells. Testosterone biosynthesis follows an enzymatic sequence of steps from *de novo* synthesized cholesterol, either intracellular cholesterol esters or extracellular supplies from circulating low-density lipoproteins. Cholesterol is converted to pregnenolone by P450-linked side-chain cleaving enzyme (P450ssc), an inner-membrane protein of mitochondria, that catalyzes the cleavage reaction. Pregnenolone may be converted to progesterone by 3β-hydroxysteroid dehydrogenase (3β-HSD), located in both mitochondria and smooth endoplasmic reticulum, or to 17α-hydroxy pregnenolone by 17α-hydroxylase/17,20-lyase (P450c17). Progesterone may be converted to 17α-hydroxy progesterone, androstenedione and finally to testosterone. Pregnenolone may be converted to 17α-hydroxy pregnenolone, dehydroepiandrosterone, androstenediol and testosterone, or it may be converted to progesterone derivates entering a different pathway.

**Figure 2:**
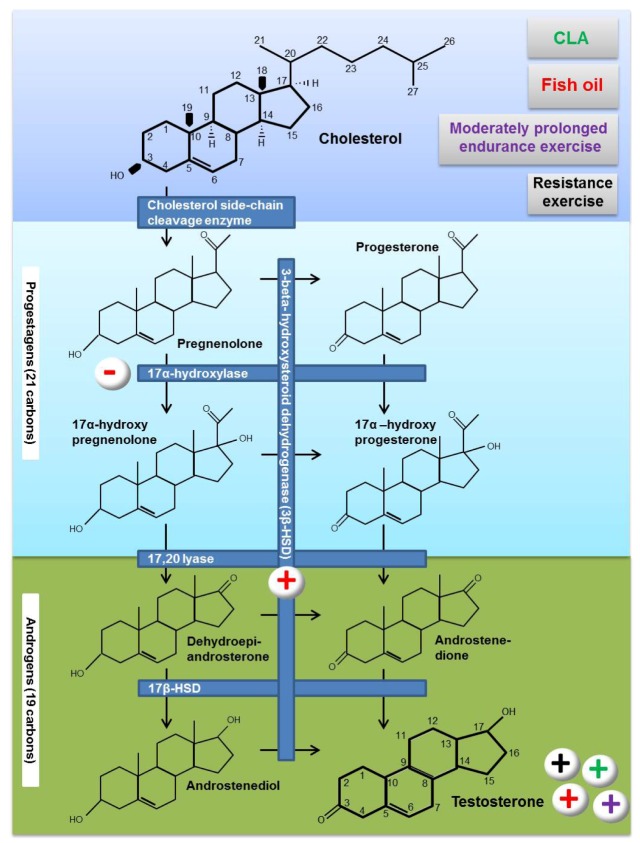
Potential effects of fat supplements and exercise on testosterone biosynthesis. The hydroxylase enzymes involved in the synthesis of testosterone have a nomenclature that indicates the site of hydroxylation (e.g., 17α-hydroxylase introduces a hydroxyl group to carbon 17) or the site of hydroxylation in addition to being identified as P450 class enzymes (e.g., the 17α-hydroxylase is also identified as P450c17). The officially preferred nomenclature for the cytochrome P450 class of enzymes is to use the prefix CYP (e.g., 17α-hydroxylase should be identified as CYP17A1). The symbols + and − indicate the over or the under expression of a specific enzyme or hormone stimulated by one of the conditions indicated with different colors (red: fish oil supplementation; green: CLA supplementation; black: resistance exercise; purple: moderate prolonged endurance exercise).

Figure 2 is a schematic representation of the enzymatic sequence of testosterone biosynthesis, starting from cholesterol. For an excellent review on testosterone biosynthesis and enzymes involved in the pathway, the reader is referred to a review by Ye *et al.* [36].

## 4. Effects of Fat Supplementation on Testosterone Biosynthesis

A new role of fat supplements is starting to be delineated in the scientific community, although the results have been mainly obtained in animals or *in vitro* studies. In fact, it has been shown that dietary fat improves reproductive performance although the molecular mechanism has not yet been elucidated. Among the different theories, there is one that hypothesizes that dietary fat may directly increase steroidogenesis [37] or directly alter the receptor composition of the testicular plasma membranes [38,39]. 

Castellano *et al.* [40], studying the effect of long-term omega-6 fatty acids supplementation on steroid production of healthy adult pigs, showed that, regardless of the EPA/DHA ratio, fish oil supplementation modified the fatty acid composition *in testis* affecting the testicular concentration of testosterone. Similar results have been obtained *in vitro* using the H295R human adrenocortical carcinoma cell line treated with cod liver oil, a model to identify chemicals that may alter steroidogenesis. This nutritional supplement derived from the liver of cod fish, like many fish oils, contains high levels of omega-3 fatty acids, EPA and DHA. 

Montano *et al.* [41] showed that exposure to extracts from cod liver oil increased the synthesis of testosterone, progesterone, estradiol and cortisol in H295R cell line. Cod liver oil also increased the expression of many genes encoding proteins involved in steroidogenesis, such as the cytochrome P450 family 1 subfamily A polypeptide 1 (CYP1A1), the melanocortin 2 receptor (MC2R), the steroidogenic acute regulatory protein (StAR), the cytochrome P450 family 11 subfamily B polypeptide 1 (CYP11B1), the cytochrome P450 family 11 subfamily B polypeptide 2 (CYP11B2), the cytochrome P450 family 19 subfamily A polypeptide 1 (CYP19A1), the cytochrome P450 steroid 17α-hydroxylase/17,20-lyase (CYP17A1), and the hydroxy-delta-5-steroid dehydrogenase 3 beta- and steroid delta-isomerase 2 (HSD3B2). A few of these genes play a key role in testosterone synthesis. StAR mediates the transfer of cholesterol from the outer mitochondrial membrane to the inner mitochondrial membrane. HSD3B2 is involved in the biosynthesis of all classes of hormonal steroids. The CYP17A1 gene, which is down-regulated, encodes the 17α-hydroxylase enzyme.

Recently, it was observed in our laboratories [32] that CLA treatment (0–7.5 μM) increased the synthesis of testosterone in a rat Leydig tumor cell line (R2C), and release in the culture media. Testosterone secretion increased linearly with CLA concentration after 48 h from treatment. In the light of this result, we investigated the level of serum testosterone immediately after an acute bout resistance exercise after three weeks of CLA supplementation (6 g/day) in trained subjects. The blood level of total testosterone after CLA supplementation following the resistance exercise bout did not increase significantly as *in vitro*, although a small increase was observed. The limitation of this study was the doses used. In fact, for the *in vivo* experiments only one dose of CLA supplementation (previously reported in the literature [24]) was administered, while it is clear that different doses and dosages need to be tested to understand the effect of CLA on testosterone synthesis.

Similar results were obtained in the ovarian tissue. It has been suggested that one of the mechanisms by which CLA may alter steroidogenesis may be by up- and down-regulating specific genes encoding for enzymes and transport proteins involved in the synthesis of prostaglandin and progesterone. In ovarian tissue, May *et al.* [42] showed that the mechanism whereby CLA improves steroidogenesis may be, in part, by decreasing prostagladin F2-α (PGF2α) synthesis in cultured bovine luteal cells through down-regulation of Cyclooxygenase-2 (COX-2) gene. No differences were observed in mRNA levels of StAR, P450scc and 3βHSD that play key roles in progesterone synthesis. 

## 5. Cellular Mechanisms Responsible for the Effect of Testosterone on Skeletal Muscle and Physical Performance

An important mechanism by which testosterone can increase the cross sectional area of the skeletal muscle fiber is the increase in the contractile protein synthesis while unaffecting protein breakdown [43]. The increase in fiber area (over 26%) is accompanied by a significant increase in the myonuclear number [44]. The major source for the addition of new myonuclei into hypertrophic muscle fibers is the satellite cells, which reside between the basal lamina and the sarcolemma of the muscle fiber [45]. The satellite cell pool, the number of resident satellite cells in the muscle, varies between individuals with different physical activity levels [46,47] and ages [48]. Moreover the satellite cell behavior (proliferation, differentiation or return to quiescence) can be affected by mechanical, local and systemic factors, such as testosterone [49]. In fact, satellite cells express androgen receptor, making the satellite cells a direct target for testosterone action [50]. Hence, another mechanism by which testosterone can induce skeletal muscle hypertrophy is by stimulating the activation of satellite cells [51] and promoting their entry into the cell cycle [52]. The cross-sectional area of a muscle (rather than volume or length) determines the amount of force it can generate by defining the number of sarcomeres which can operate in parallel. 

The effects of testosterone on human performance have been the objective of studies since the early 1980s [53], but only in the last decade have more carefully designed studies been conducted, although all of these studies investigated the effect of exogenous testosterone [54]. It was demonstrated that supraphysiological doses of testosterone enhance maximal voluntary strength by increasing muscle mass and not by changing contractile properties, and the improvement in strength was dose-dependent [55]. Rogerson *et al.* [56] observed an increase in output of work and power during cycle sprinting in subjects integrated with testosterone. The data suggest that testosterone may increase sprinting performance in humans as in animals [57]. Moreover, testosterone may improve performance in sprint events by reducing reaction time, since it has been shown that testosterone regulates neuromuscular transmission in rats [58,59]. It has been hypothesized that testosterone may affect endurance performance since it induces an increase in hemoglobin concentration and hematocrit. This hypothesis was confirmed only in animals; in fact, exogenous testosterone increases endurance performance in rats [60], while in humans it does not improve performance [54]. The difference between species may be explained by the diversity in the relative proportion of type I fibers available for enhancement [61]. No changes in the ability of a muscle to continue in performing an exercise (fatigability) have been observed after testosterone use in humans [55].

## 6. Implications

Testosterone is a steroid hormone with anabolic and anticatabolic effect on muscle tissues, playing a critical function for muscle gain and muscle performance of athletes [62,63]. It has been demonstrated that the acute increase in serum concentration of testosterone after resistance exercise depends on exercise program variables (intensity, volume, duration, rest, muscle mass trained) and individual characteristics (age, health, fitness level) [64,65], while it has been shown that moderately prolonged endurance exercise induces an increase in the concentration of free testosterone mediated by a sympathetic stimulation of the testicles [66,67]. 

Differently from fat supplements, the fat ingested with the daily diet may have the potential to alter the regulation and metabolism of testosterone in athletic men [68]. Volek *et al.* [68] showed that MUFA (mono-unsaturated fatty acid) and SFA (saturated fatty acid) were the strongest predictors of circulating testosterone in healthy athletic men during rest, while there was no significant correlation between PUFA and testosterone, there was a significant negative correlation between PUFA/SFA ratio and testosterone levels. The concept that high fat diets lead to alterations in serum level of testosterone of athletes, has been shown also by different research groups [69]. Taking into consideration the fact that athletes may experience a decline in testosterone concentrations due to overtraining and very low fat diets (this condition may be aggravated in athletes of specific sports, such as gymnasts, wrestlers and boxers), fish oil and CLA supplementation may be proposed to compensate the alteration in serum testosterone induced by prolonged and intense exercise training period (over training). These supplements may also promote an anabolic environment over a training program. 

On the other hand, fat supplement side-effects have never been demonstrated and documented. If fat supplements induce an increase in blood testosterone, this may have an effect on several other tissues, among which include stem or progenitor cells [70]. Testosterone has been reported to have a pro-survival and growth-stimulatory effect on mature progenitor cells [71] or a negative effect on the cardiovascular system down-regulating signal transducer and activator of transcription 3 (STAT3) and suppressor of cytokine signaling 3 (SOCS3) expression during acute ischemia and reperfusion [72]. Hence, indirectly, fat supplements may have an effect on cardiac progenitor cells which are fundamental during heart development [73,74], myocardium homeostasis and myocardium regeneration [75]. This consideration is very important taking into account that cardiovascular diseases are the leading causes of death among athletes [76].

Additional research on the effect of fish oil and CLA supplementation on enzymes leading to testosterone synthesis are important to clarify the molecular mechanisms by which fat supplements may contribute to increase the anabolic effect of exercise, and the side-effects of this kind of supplementation. 

## Abbreviations

CLA: conjugated linoleic acid
DHA: docosahexaenoic acid
EPA: eicosapentaenoic acid
c9: cis-9
t11: trans-11
t10: trans-10
c12: cis-12
RBC: red blood cells
PUFA: polyunsaturated fatty acids
P450ssc: P450 side-chain cleavage enzyme
3β-HSD: 3β-hydroxysteroid dehydrogenase
P450c17: 17α-hydroxylase/17,20-lyase
17β-HSD: 17β-hydroxysteroid dehydrogenase
CYP17A1: cytochrome P450, steroid 17α-hydroxylase/17,20-lyase
H295R: human adrenocortical carcinoma cells
PGF2α: prostagladin F2-α
MUFA: mono-unsaturated fatty acid
SFA: saturated fatty acid
CYP1A1: cytochrome P450, family 1, subfamily A, polypeptide 1
MC2R: melanocortin 2 receptor
StAR: steroidogenic acute regulatory protein
CYP11B1: cytochrome P450, family 11, subfamily B, polypeptide 1
CYP11B2: cytochrome P450, family 11, subfamily B, polypeptide 2
CYP19A1: cytochrome P450, family 19, subfamily A, polypeptide 1
HSD3B2: hydroxy-delta-5-steroid dehydrogenase, 3 beta- and steroid delta-isomerase 2
STAT3: signal transducer and activator of transcription 3
SOCS3: suppressor of cytokine signaling 3
COX-2: cyclooxygenase-2
